# Cancer model with moving extinction threshold reproduces real cancer data

**DOI:** 10.1098/rsif.2024.0844

**Published:** 2025-10-01

**Authors:** Frank Bastian, Hassan Alkhayuon, Kieren Mulchrone, Micheal O'Riordain, Sebastian Maciej Wieczorek

**Affiliations:** ^1^ School of Mathematical Sciences, University College Cork, Western Road, Cork T12 XF62, Ireland; ^2^ Department of Surgery, Mercy University Hospital, Cork, County Cork, Ireland

**Keywords:** cancer development, mathematical model, extinction threshold, noise-induced tipping, Allee effect, breast cancer risk

## Abstract

We propose a simple dynamic model of cancer development that captures carcinogenesis and subsequent cancer progression. A central idea of the model is to include the immune response to cancer, which leads to the emergence of an extinction threshold. We first identify the limitations of commonly used extinction threshold models from population biology in reproducing typical cancer progression. We then address these limitations by deriving a new model that incorporates: (i) random mutations of stem cells at a rate that increases with age and (ii) immune response whose strength may also vary over time. Our model accurately reproduces a wide range of real-world cancer data: the typical age-specific cumulative risk of most human cancers, the progression of breast cancer in mice and the unusual age-specific cumulative risk of breast cancer in women. In the last case, we model the different immune response at different phases of the menstrual cycle and menopausal treatment and show that this leads to a moving extinction threshold. This approach provides new insights into the effects of hormone replacement therapy and menstrual cycle length on breast cancer in women. More generally, it can be applied to a variety of other cancer scenarios where the immune response or other important factors vary over time.

## 1. Introduction

Cancer is one of the leading causes of death worldwide, accounting for 10 million fatalities a year [1]. The global lifetime risk of developing any type of cancer is estimated to be approximately 25%; however, this varies significantly with socioeconomic factors encapsulated in the Human Development Index [2]. Additionally, cancer cases are forecast to rise by 77% between 2022 and 2050 [3] due to several factors, including changing demographic profiles in developed countries and improving living standards in underdeveloped countries. The spectre of an increasing cancer burden is a cause for concern in both economic and social terms [1,4]. There is an urgent need to better understand the critical points of cancer development and to improve cancer treatment [5]. This is evidenced by several initiatives such as the War on Cancer in the USA [6], or Europe’s Beating Cancer Plan [7].

Some cancers develop in unusual ways. For example, breast cancer is the most common cancer in the USA, with 13.1% of women expected to develop it during their lifetime [8]. In these women, the age-specific cumulative risk appears to increase polynomially up to the age of menopause and linearly thereafter^1^; see also [9,10]. This is quite unusual for cancer, given that the cumulative risk of other common cancers, such as lung, blood or colorectal cancer, increases exponentially ([11], section 4). This raises important research questions about the dynamic mechanisms underlying the development of different cancers. To address this question, we categorize cancers into: (i) *typical cancers*, which have an exponential-like cumulative risk (the vast majority of cancers), and (ii) *atypical cancers*, which have an unusual non-exponential cumulative risk (e.g. breast cancer in women).

Over the last century, several effective treatment therapies have been developed e.g. surgery, chemotherapy and radiation [12]. These treatments are often applied aggressively, at the maximum tolerated dose [13], in an effort to maximize the probability of cure. However, aggressive treatment strategies may fail in the long term. The maximum tolerated dose eliminates sensitive cancer cells, allowing treatment-resistant cancer cells to flourish in the aftermath [14]. This may render the treatment ineffective. Alternative treatment strategies have been proposed that apply lower drug doses in an optimal way [15,16] to maintain a balance between non-resistant and resistant cancer cells to prolong patient survival [13,17]. To test and validate novel treatment strategies, a robust, reliable and accessible mathematical description of cancer development is required.

Existing mathematical models of cancer development vary in complexity. While complex models can be more realistic, simple dynamic models make it easier to understand the critical points of cancer development and identify optimal treatment strategies. This article presents a simple dynamic model that is process-based, captures the key mechanisms and critical points of cancer development and reproduces a wide range of real cancer data.

For example, Markov chain models and agent-based models (ABMs) are considered to be well suited to study evolutionary and ecological processes at different temporal and spatial scales [18–20]. ABMs are very flexible because biological observations can be translated directly into simulation rules [21,22]. However, it is often challenging to choose sensible parameters and validate ABMs [23,24]. Furthermore, the physical scale of simulations is constrained by available computational resources [25]. The use of dynamic ordinary, partial and stochastic differential equation models circumvents most of these drawbacks [26–28]. In the simple models, the changing number of cancer cells in a tumour is described using population growth models, such as the logistic [29,30] or Gompertzian model [13]. However, while describing the progression of cancer quite well, such models lack a threshold separating growth from natural extinction, known as the strong Allee effect in population biology [31]. In other words, typical growth models predict that, no matter how small the initial cluster of cancer cells, a large tumour is the inevitable outcome. This is inconsistent with the observed processes where cancer cells, or their precursors, continuously develop and are eliminated naturally by immune suppression mechanisms [32,33].

In this article, we propose a novel model of cancer initiation and growth. We show that the model is consistent with a wide range of available data on cancer growth and age-specific incidence rates at the population level. We include in the model the response of the immune system to mutations, which normally prevents cancer from developing. This naturally leads to the emergence of an extinction threshold. We also show that the threshold need not be fixed. It can change over time, for example, due to a changing immune response, resulting in a moving extinction threshold. We then develop a simple stochastic model of cell mutations and combine it with the extinction threshold growth model. In this way, we are able to reproduce the exponential-like increase in age-specific cumulative risks of typical cancers using a constant immune response. We then go one step further and reproduce the unusual non-exponential increase in the age-specific cumulative risk of breast cancer in women. To achieve this, we use a time-varying immune response that reflects the different progesterone levels at different phases of the menstrual cycle and menopausal treatment. In this way, we provide new insights into the dynamic mechanisms that underlie the development of different cancers.

This article is organized as follows. In §2, we discuss the importance of an extinction threshold in cancer development, review the classical models of population growth with an extinction threshold and show their limitations in accurately describing cancer development. We then propose a new dynamic model that overcomes the limitations of the classical models. In §3, we demonstrate very good agreement between the proposed model and available data on untreated breast cancer progression in mice, typical age-specific cumulative risk of colorectal cancer in women and unusual age-specific cumulative risk of breast cancer in women. The last result is achieved by consecutively including processes related to the menstrual cycle, menopause and postmenopausal hormone replacement therapy (HRT). Finally, new insights into cancer development derived from the proposed model are highlighted in §4.

## 2. A simple dynamic model of cancer development

The aim of this section is to develop a simple dynamic model that: (i) captures two phases of cancer development, the onset of cancer (carcinogenesis) and its subsequent progression, (ii) is formulated in terms of cell birth and death processes alone for clear biological interpretation, and (iii) shows very good agreement with real cancer data.

We discuss the limitations of classical population growth models in capturing both phases of cancer development and propose a new model that overcomes these limitations. One of the central concepts that emerges from our model is an *extinction threshold.* In the context of cancer development, this threshold is a critical size of a cluster of mutated cells below which the cluster can be eradicated by the immune system alone and above which the cluster develops into cancer.

### 2.1. Importance of an extinction threshold

**Figure 1.**
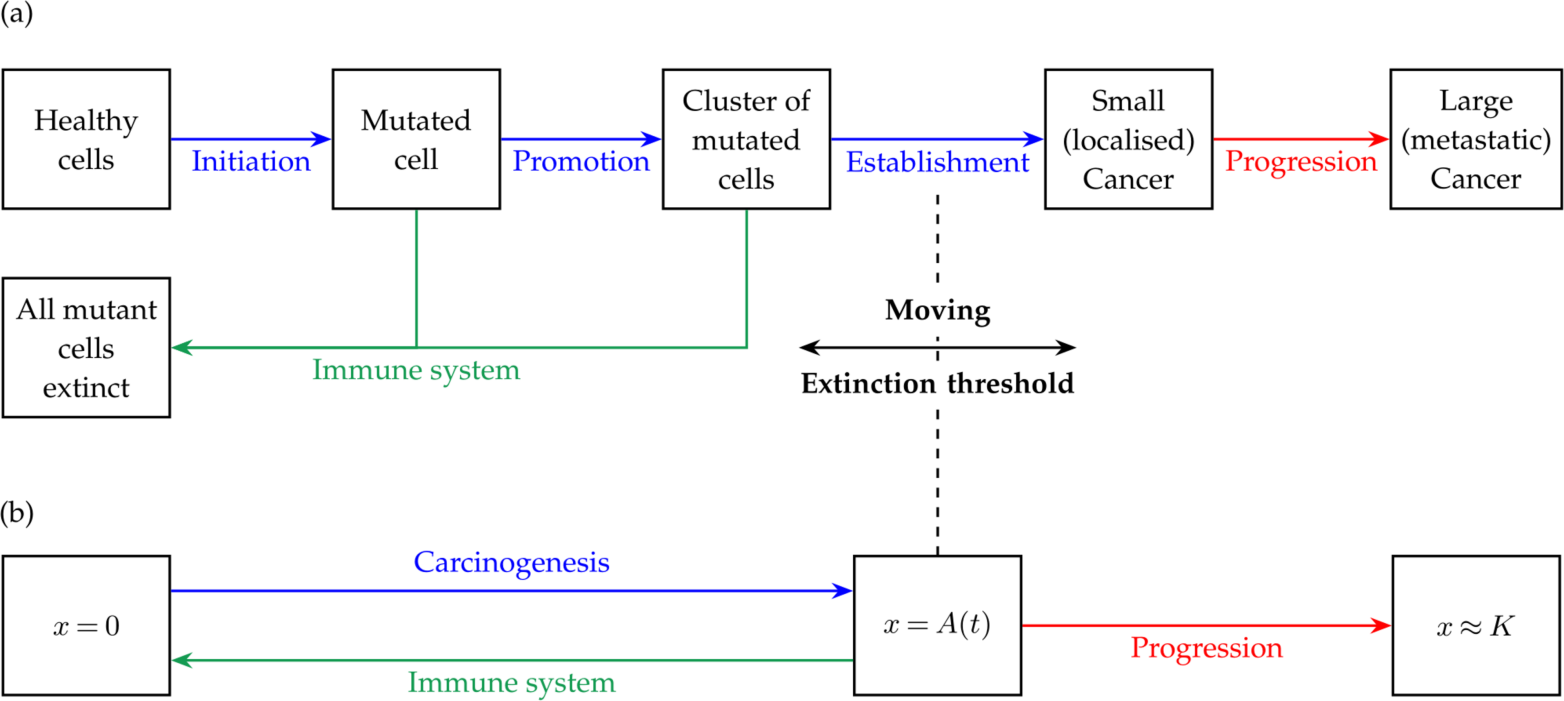
Two conceptual diagrams of cancer development, including (blue) carcinogenesis and (red) subsequent cancer progression. In (a), carcinogenesis is depicted as a three-step process. It consists of initiation, promotion and establishment, with a moving extinction threshold. In (b) carcinogenesis is simplified to a one-step process with a moving extinction threshold A(t) for the sake of the simple dynamic model developed in §2.

Carcinogenesis can be described in terms of a three-step process consisting of *initiation*, *promotion* and *establishment*, as shown in figure 1a.

*Initiation* occurs when a healthy cell with the ability to divide suffers a mutation that is beyond repair. A *cell mutation* is a damage to the DNA sequence of a cell. Mutations occur naturally as a result of oxidation, ionizing radiation, UV radiation, smoking, chemicals in food and drink and other factors. It is estimated that, on average, a single cell can suffer up to $10^{5}$ instances of DNA damage per day from internal (endogenous) and external (exogenous) sources [34]. This raises the question of why carcinogenesis is comparatively rare and what causes only certain mutations to develop into cancer. Typically, genes such as tumour suppression genes or DNA mismatch repair genes repair damaged cells or initiate programmed death (apoptosis) of unwanted cells, greatly limiting the effects of mutations [35–37]. However, if the repair mechanisms fail, the mutated cell undergoes promotion, which occurs when the single mutated cell rapidly divides (proliferates) to form a small cluster of mutated cells [32]. Although the mutation is irreversible at this stage, the immune system may still be able to eliminate such clusters through a variety of mechanisms [38,39]. Crucially for our model, this elimination is only possible if the cluster is small enough and the immune response is strong enough [32]. *Establishment* occurs when the immune system fails to eliminate the cluster and the cluster develops into cancer. In other words, there is an *extinction threshold* above which a cluster of mutated cells can no longer be eradicated by the immune system alone. This is evidenced, for example, by the work of Pitot and Dragan [32] and Kirsch-Volders *et al.* [33], who identify a ‘threshold’ separating promotion from progression in cancer growth data. In a model, such a threshold may be introduced ad hoc [40] or may emerge from the physical processes being considered [22].

In the following subsections, we will progressively construct a simple dynamic model of cancer development that is based on the conceptual diagram in figure 1b, incorporates the key physical processes and has an emergent (moving) extinction threshold.

### 2.2. Cancer progression with and without an extinction threshold

We start with the second phase of cancer development, progression above the extinction threshold, where small cancers grow in size and potentially spread to other organs (metastasis). We begin with a brief review of classical growth models from population biology with no extinction threshold. We then introduce a defining feature of cancer progression to highlight limitations of classical growth models in capturing cancer progression in the presence of an extinction threshold. Finally, we propose a new model that overcomes the limitations of classical growth models.

The simplest model of population growth with no extinction threshold, proposed by Thomas Malthus [41], assumes unbounded *exponential growth*. A more realistic model, proposed by Pierre–François Verhulst [42], accounts for resource limitation and introduces bounded *logistic growth* towards a *carrying capacity* K—the maximum population size that can be supported by the environment. A specific feature of logistic growth is the *symmetric progression*: growth of small populations and saturation of large populations as they approach carrying capacity K occur at the same rate. While the progression of certain cancers can be described by the symmetric logistic growth model [29,43], many known cancers exhibit *asymmetric progression*, where small populations grow faster than large populations saturate as they approach K [44,45]. These cancers are often described by the asymmetric growth model proposed by Benjamin Gompertz [46]. As cancer patients die before the tumour reaches its carrying capacity, cancer development data approach but never reach K.

#### 2.2.1. A defining feature of cancer progression

We use the asymmetry of cancer progression as its *defining feature,* quantified by the ratio of the growth rate of small populations, $\lambda_{\text{grow}}$, to the saturation rate of large populations at K, $\lambda_{\text{sat}}$. To the best of our knowledge, there are no examples of cancer progression where small populations grow more slowly than large populations saturate at K. Thus, to reproduce typical cancer progression, the defining feature must satisfy [29,43–45],


(2.1) $$\left|\frac{\lambda_{\text{grow}}}{\lambda_{\text{sat}}}\right|\geq 1.$$

**Figure 2.**
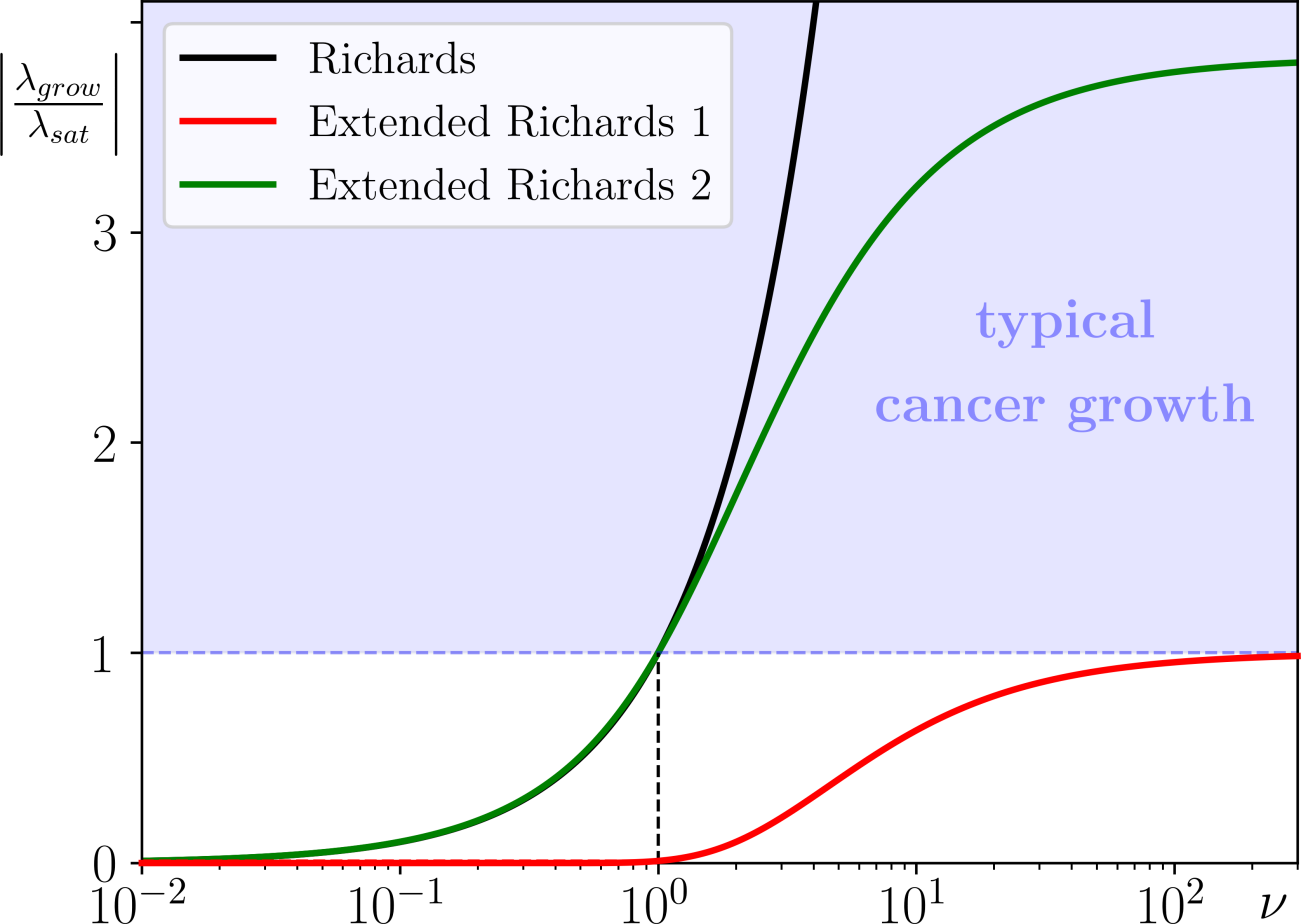
The defining feature of cancer progression (the ratio of the small cancer's growth rate $\lambda_{\text{grow}}$ to the large cancer's saturation rate $\lambda_{\text{sat}}$) versus the growth parameter ν for: (black) the Richards model with no extinction threshold in equation (2.2), (red) the classical extended Richards model 1 with an extinction threshold in equation (2.3) and (green) the proposed extended Richards model 2 with an extinction threshold in equation (2.4). r=1, A=1 and K=100. (In equation (2.4), μ and s have been adjusted for each value of ν to keep A=1 and K=100). Note the logarithmic scale for ν.

This requirement is represented in figure 2 by the blue region. The interior of the blue region corresponds to asymmetric progression in which small populations grow faster than large populations saturate at K, while the (dashed) lower boundary, $|\lambda_{\text{grow}}/\lambda_{\text{sat}}|=1$, corresponds to the symmetric progression.

#### 2.2.2. Richards’ model: a classical growth model with no extinction threshold

We will be interested in a more general growth model, giving rise to the so-called *generalized logistic growth*, proposed by Francis John Richards [47]. A particular advantage of Richards’ model is that it can be parameterized to give different shapes of temporal growth, both symmetric and asymmetric, making it very versatile for reproducing the progression of different types of cancer. We denote the number of cancer cells by a continuous variable x(t)≥0, and write Richards’ model as an explicitly solvable ordinary differential equation (ODE),^2^


(2.2) $$\frac{\text{d}x}{\text{d}t}=\nu rx-\nu \mu x^{\frac{\nu +1}{\nu }}=\nu rx\left(1-{\left(\frac{x}{K}\right)}^{\frac{1}{\nu }}\right),$$

where t is time, dx/dt is the instantaneous rate of change of the number of cancer cells, νr>0 is a constant per capita growth rate, $\nu \mu x^{1/\nu }\geq 0$ is a population-dependent per capita death rate that increases with x, ν>0 is the shape parameter and $K=(r/\mu {)}^{\nu }$ is the carrying capacity.^3^ Other general models of cancer growth include, for example, the von Bertalanffy model [48]. We now describe the properties of this model and refer to ([11], section 1.1) for technical details.

The Richards model has two stationary (fixed in time) solutions, also called equilibrium points,


x=0 and x=K.

Linear stability analysis reveals that the extinction x=0 is exponentially unstable with divergence rate $\lambda_{\text{grow}}=\nu r$, while the carrying capacity x=K is exponentially stable with convergence (saturation) rate $-\lambda_{\text{sat}}=r$. In other words, small populations x(t) in equation (2.2) grow exponentially at the rate νr, while large populations saturate exponentially at the rate r as they approach K.

Thus, the defining feature of cancer progression in this model is


$$\left|\frac{\lambda_{\text{grow}}}{\lambda_{\text{sat}}}\right|=\nu .$$

Setting ν=1 gives the Verhulst model with symmetric logistic growth, whereas setting ν>1 gives a model with the desired asymmetric progression. The Gompertz model is obtained in the limit ν→∞. In other words, the defining feature of cancer progression in Richards’ model (2.2), represented by the black curve in figure 2, falls within the desired blue region when ν≥1.

#### 2.2.3. Extended Richards model 1: a classical growth model with an extinction threshold

A simple model of population growth with an extinction threshold was proposed by Vito Volterra [49], who modified Verhulst’s model. In our first attempt to introduce an extinction threshold, we modify Richards’ model (2.2) in a similar way. We refer to this model as the *extended Richards model 1 (ERM1),*


(2.3) $$\begin{array}{rl} \frac{\text{d}x}{\text{d}t} & =-\nu^{2}r\;x+\nu^{2}\mu \;x^{\frac{\nu +1}{\nu }}-\nu^{2}\gamma \;x^{\frac{\nu +2}{\nu }}\\ & =-\nu^{2}r\;x\left(1-{\left(\frac{x}{A}\right)}^{\frac{1}{\nu }}\right)\left(1-{\left(\frac{x}{K}\right)}^{\frac{1}{\nu }}\right), \end{array}$$

where t is time, dx/dt is the instantaneous rate of change of the number of cancer cells, $\nu^{2}r>0$ becomes a constant per capita death rate of small populations, $\nu^{2}\mu x^{1/\nu }\geq 0$ becomes a population-dependent per capita birth rate that increases with x, and ν>0 is the shape parameter. The third term is new and corresponds to a population-dependent per capita death rate of large populations, $\nu^{2}\gamma x^{2/\nu }\geq 0$, which also increases with x. We now describe the properties of ERM1 and refer to ([11], section1.2) for technical details.

For a suitable choice of the new parameter γ, which quantifies the death rate of large populations, ERM1 has three equilibrium points,


x=0,x=A and x=K,

including an extinction threshold 0<A<K, given by


$$\begin{array}{c} A={\left(\frac{\mu -\sqrt{\mu^{2}-4r\gamma }}{2\gamma }\right)}^{\nu }. \end{array}$$

The most commonly used version of ERM1, obtained by setting ν=1 in equation (2.3), is the original Volterra model [49]. On the other hand, in the limit ν→∞, we recover a less commonly used version of ERM1, namely the Gompertz model with an extinction threshold [50].

**Figure 3.**
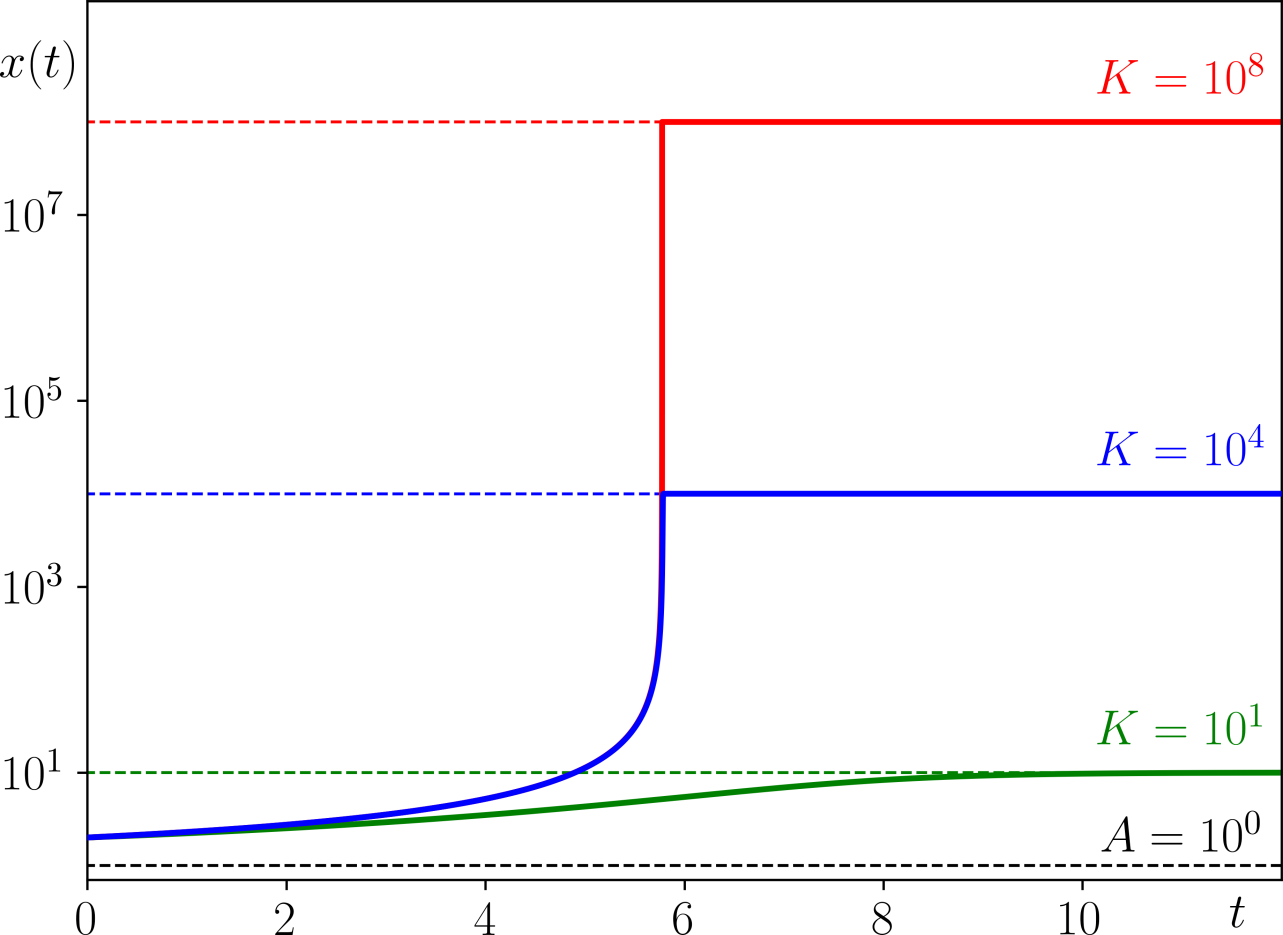
Population growth x(t) in the commonly used version of ERM1 (equation (2.3) with ν=1) started from x(0)=2 with a per capita decay rate r=0.12, extinction threshold A=1 and three different values of carrying capacity: (green) K=10 , (blue) $K=10^{4}$ and (red) $K=10^{8}$. For realistic values of K, the solutions show an unusual step-like behaviour that does not resemble the progression of any known cancer. For $K=10^{4}$, the (blue) progression curve takes approximately 5.5 time units to reach 1% of K, and only $10^{-1}$ time units to grow from 1% to 99% of K. For $K=10^{8}$, the (red) progression curve takes a similar time to reach 1% of K and only $10^{-5}$ time units to grow from 1% to 99% of K. Such population jumps are also unusual in ecology [40]. Note the logarithmic scale for x(t).

Linear stability analysis reveals that the extinction threshold x=A is exponentially unstable with divergence rate $\lambda_{\text{grow}}$, and the carrying capacity x=K is exponentially stable with convergence (saturation) rate $-\lambda_{\text{sat}}$. However, the introduction of an extinction threshold by this approach has major limitations in reproducing typical cancer progression. To formulate these limitations rigorously, we prove in ([11], section 1.2) that, in ERM1 with extinction threshold A, small populations always grow more slowly than large populations saturate as they approach K. In other words, the defining feature of cancer progression in ERM1,


(2.4) $$\begin{array}{r} \left|\frac{\lambda_{\text{grow }}}{\lambda_{\text{sat }}}\right|={\left(\frac{A}{K}\right)}^{\frac{1}{\nu }}<1\;\text{ for all }\;\nu >0 \end{array},$$

is in disagreement with the progression of known cancers. This is further illustrated in figure 2, where the defining feature of cancer progression in ERM1, represented by the red curve, does not overlap with the desired blue region for any ν>0. Moreover, in typical cancer development, A is several orders of magnitude smaller than K. Thus, if we fix ν=1 (the most commonly used Volterra model), small populations grow several orders of magnitude slower than large populations saturate at K; the red curve in figure 2 is nowhere near the desired blue region when ν=1. This has serious implications for the shape of the temporal cancer progression curve. The populations stay just above A for a long time before approaching K sharply, in a somewhat unrealistic step-like behaviour shown in figure 3. This behaviour can be further explained by looking at the limit of large K, where $\lambda_{\text{grow}}=r(1-A/K)$ tends to r and $-\lambda_{\text{sat}}=r(K/A-1)$ tends to ∞ ([11], section 1.2).

At best, when ν→∞ for a fixed K (the less commonly used Gompertz model with an extinction threshold), the defining feature $|\lambda_{\text{grow}}/\lambda_{\text{sat}}|$ approaches 1 from below and progression becomes symmetric; the red curve in figure 2 approaches the desired blue region from below for large ν.

In summary, a different approach to an extinction threshold is required to achieve the desired asymmetry (2.1) that is characteristic of typical cancer progression.

#### 2.2.4. Extended Richards model 2: a new growth model with an extinction threshold

In this section, we construct a new model of cancer progression that builds on the strengths of the classical growth models and overcomes their limitations. We aim to retain: (i) an extinction threshold, (ii) the versatility of growth above the extinction threshold, ranging from symmetric to the desired asymmetric progression in line with (2.1), and (iii) formulation in terms of birth and death processes alone for clear biological interpretation.

Guided by the results of the previous section and the points above, we start with the Richards model (2.2) and introduce a new process that accounts for cancer suppression mechanisms. In this process, the immune system prevents or eliminates a certain number of mutated cells per day.^4^ We represent this process by an additional cell death term—s. Furthermore, we recognize that the immune response will vary in time t, possibly in a complicated way and on different time scales, e.g. due to seasonal variations, viral infections, the menstrual cycle, immune deficiencies, immunotherapy, ageing, etc. We, therefore, allow the additional term —s(t) to vary over time. This means that the immune system prevents or eliminates a certain number of mutated cells each day, and this number changes over time as different factors influence the strength of the immune system. This gives us the *extended Richards model 2 (ERM2)*,


(2.5) $$\begin{array}{r} \frac{\text{d}x}{\text{d}t}=\left\{ \begin{array}{ll} \nu rx-\nu \mu x^{\frac{\nu +1}{\nu }}-s(t) & \text{ if }x>0,\\ 0 & \text{ if }x=0, \end{array}\right. \end{array}$$

where the second equation keeps x(t) non-negative and thus physically relevant. Here, t is time, dx/dt is the instantaneous rate of change of the number of cancer cells, νr>0 is a constant per capita growth rate, $\nu \mu x^{1/\nu }\geq 0$ is a population-dependent per capita death rate that increases with x, and ν>0 is the shape parameter. We now describe the properties of ERM2 and refer to ([11], section 1.3) for technical details.

Owing to the time-varying s(t), the non-autonomous ERM2 has only one equilibrium point, namely extinction x=0. This equilibrium is always stable and, in contrast to ERM1, the population x(t) becomes extinct in finite time.

To gain further insight into the behaviour of ERM2, it is useful to start with a simplified description, where the immune response is constant (fixed in time), that is s(t)=s=const. Then, for a suitable choice of s, ERM2 has three equilibrium points,


(2.6) $$\begin{array}{r} x=0,\;x=A\;\text{ and }\;x=K, \end{array}$$

where 0<A<K. While there is no closed-form formula for A, the position of the *stationary extinction threshold* $A∼10^{a}$ can be approximated by


(2.7) $$\begin{array}{lcr} & A\approx \frac{s}{\nu r-\mu (\nu +a\;\;\ln\;10)}. & \end{array}$$

This approximation allows us to identify what we call *threshold parameters*: the immune response s, the per capita growth rate νr and the death rate parameter νμ. It also shows how the position of the extinction threshold changes with different threshold parameters.

Linear stability analysis reveals that the stationary extinction threshold x=A is exponentially unstable with divergence rate $\lambda_{\text{grow}}$, and the carrying capacity x=K is exponentially stable with convergence (saturation) rate $-\lambda_{\text{sat}}$. Most importantly, we prove in ([11], section 1.3) that small populations grow faster than large populations saturate as they approach K if the growth parameter ν is set greater than one. To be specific, the defining feature of cancer progression in ERM2,


$$\left|\frac{\lambda_{\text{grow }}}{\lambda_{\text{sat }}}\right|\geq 1\;\text{ for all }\;\nu \geq 1,$$

is in agreement with the progression of known cancers. This is further illustrated in figure 2, where the defining feature of cancer progression in ERM2 with a stationary extinction threshold, represented by the green curve, is in the desired blue region for ν≥1.

A more realistic description will consider how certain threshold parameters vary over time. This means that the threshold will also vary over time and one will have to consider a *moving extinction threshold* A(t). Our focus will be on a moving extinction threshold due to a time-varying immune response s(t).

In summary, ERM2 incorporates an extinction threshold in a way that is process-based, preserves the versatility of different growth functions and, most importantly, retains the progression characteristics of typical cancers.

### 2.3. Carcinogenesis: random cell mutations

We now consider the first phase of cancer progression, carcinogenesis, during which the tumour is initiated. Crucially, we model carcinogenesis as an interplay between random cell mutations and the extinction threshold. We consider a fixed number of healthy stem cells n. We then assume that there is a small probability p(t) that each healthy stem cell will mutate in one day [51]. In other words, the mutation of a single cell is a *Bernoulli trial* [52] with a small probability of success (mutation) p(t). If we additionally assume that the mutations of individual cells are independent events, the mutation of a group of n cells is a *binomial process* [53]. In such a process, the probability P that m of n healthy cells will mutate in one day is given by the binomial distribution.


(2.8) $$\begin{array}{lcr} & P\left(m;n,p(t)\right)=\frac{n!}{m!(n-m!)}\;\;p(t{)}^{m}\;{\left(1-p(t)\right)}^{\left(n-m\right)}, & \end{array}$$

with expected value np(t) and variance np(t)(1−p(t)).

An important aspect of our model is that the probability p(t) that a single cell will mutate in one day evolves slowly over a lifetime. We assume that p(t) starts from $p(0)=p_{0}$ at birth, increases linearly with time t,


(2.9) $$\begin{array}{lcr} & p(t)=p_{0}\left(1+\frac{\delta \;t}{100\;T}\right), & \end{array}$$

and reaches a δ per cent increase at life expectancy T. This means that the binomial distribution in (2.8) also evolves over a lifetime, as shown in ([11], figure 1).

Our model of carcinogenesis can be understood in terms of two competing processes. On the one hand, random cell mutations lead to clusters of mutated cells. On the other hand, the immune system tries to eradicate these clusters, and its strength is represented by the position of the extinction threshold. Meanwhile, the mutation rate increases slowly with age, and the extinction threshold moves over time to reflect, for example, changes in the strength of the immune system. Whether a cluster of mutated cells can be eliminated by the immune system alone or develops into a small cancer depends on its size relative to (i.e. below or above) the current position of the extinction threshold.

### 2.4. The complete cancer development model

In this section, we construct a *complete model of cancer development* by combining the ERM2 of cancer progression with an extinction threshold, proposed in §2.2.4, with the random mutation model, proposed in §2.3.

First, we rewrite ERM2 in equation (2.5) as a stochastic differential equation with a noisy mutation term dξ,


$$\begin{array}{r} \text{d}x=\left\{ \begin{array}{ll} \left(\nu rx-\nu \mu x^{\frac{\nu +1}{\nu }}-s_{m}(t)\right)\text{d}t+\text{d}\xi & \text{ if }x>0,\\ 0 & \text{ if }x=0, \end{array}\right. \end{array}$$

where $s_{m}(t)$ is the augmented immune response in the presence of mutations.^5^ Next, we fix a short time interval Δt and use the Euler–Maruyama method to approximate solutions to the stochastic differential equation with x>0 by a stochastic difference equation,


$$\begin{array}{c} \begin{array}{rl} x(t+\Delta t)=x(t) & +\left[\nu rx(t)-\nu \mu x(t{)}^{\frac{\nu +1}{\nu }}-s_{m}(t)\right]\Delta t\\ & +\left(\xi (t+\Delta t)-\xi (t)\right), \end{array} \end{array}$$

where the random variable (ξ(t+Δt)−ξ(t)) is the number of new mutations in the time interval Δt. We then set Δt=1 day in the equation above and define^6^


$$\begin{array}{c} F(x,t):=x(t)+\left[\nu rx(t)-\nu \mu x(t{)}^{\frac{\nu +1}{\nu }}-s_{m}(t)\right]+m(t), \end{array}$$

with new mutations in 1 day,


m(t)=ξ(t+1)−ξ(t),

obtained from the probability distribution in equation (2.8). Finally, we write our complete cancer development model in terms of F(x,t) as a non-autonomous stochastic difference equation with a time step interval of 1 day,


(2.10) $$\begin{array}{lcr} & \begin{array}{r} x(t+1)=\left\{ \begin{array}{cc} F(x,t) & \text{if }\;F(x,t)\geq 0,\\ 0 & \text{if }\;F(x,t)<0. \end{array}\right. \end{array} & \end{array}$$

The instantaneous position of the *moving extinction threshold* $A(t)∼10^{a}$ in the complete model (2.10) can be approximated by


(2.11) $$\bar{A}(t)\approx \frac{s_{m}(t)-\bar{m}(t)}{\nu r-\mu (\nu +a\;\ln10)},$$

where m(t) is the time average of m(t) over a chosen time interval during which $s_{m}(t)$ does not change significantly; see ([11], section 1.5) for more details. This approximation shows that the extinction threshold for the complete model can move over time due to the randomly varying mutations, m(t), or a combination of m(t) and the changing immune response, $s_{m}(t)$.

An interesting consequence of a moving extinction threshold is that if the cluster of mutated cells x(t) exceeds the moving threshold $\bar{A}(t)$, there are two possible scenarios. Often, the cluster of mutated cells remains above the moving threshold and progresses directly into cancer. However, there is another possibility, known as the rescue event [54]: the threshold rises faster than the growth of the cluster of mutated cells, the cluster is soon back below the moving threshold, and the transition to cancer is avoided; see ([11], section 1.5) for more details.

**Figure 4.**
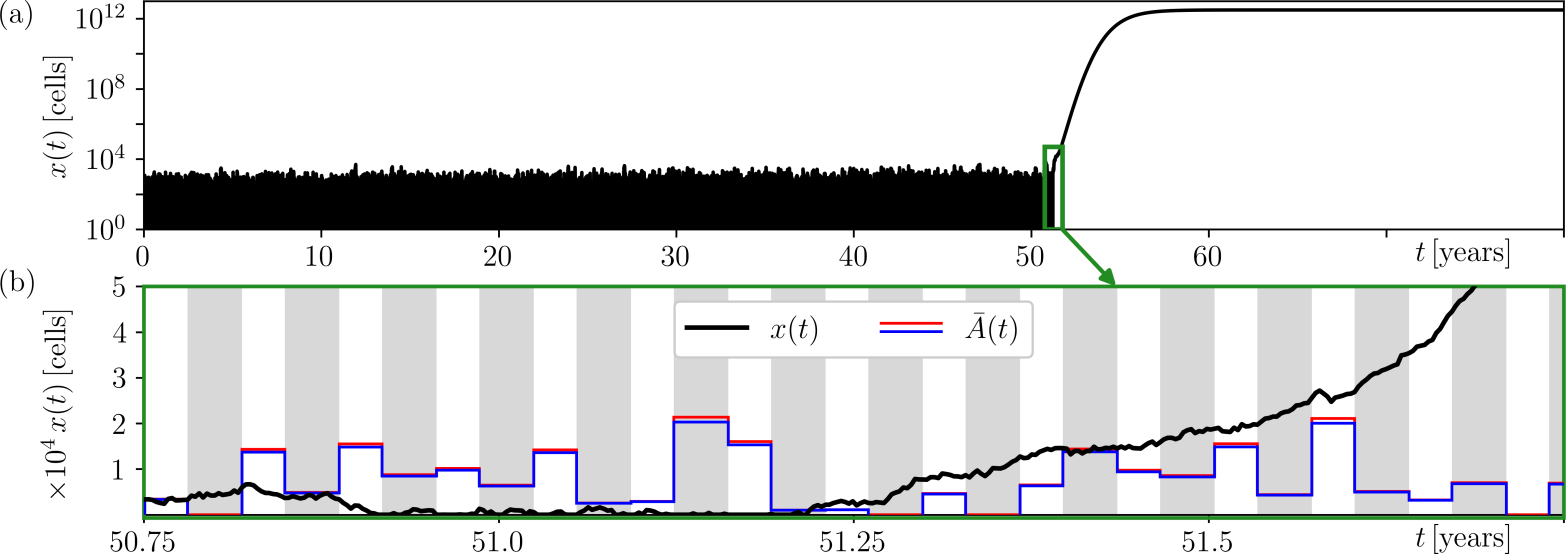
(a) An example of breast cancer development x(t) in the complete model (2.10) for a single realization of random mutations m(t) using the parameter values in ([11], table 3), and for periodic variations in the immune response $s_{m}(t)$ due to menstrual cycle in equation (3.1). (b) An extended view of the critical point around age 51.25, when the (black) number of mutated cells x(t) exceeds the (blue/red) moving extinction threshold $\bar{A}(t)$ and develops into cancer. $s_{m}(t)$ takes values: (grey stripes) $s_{\text{min}}=np_{0}+262$ cells day^−1^ during the 14 day long luteal phases with a weaker immune response, and (white stripes) $s_{\text{max}}=np_{0}+354$ cells day^−1^ during the 11 day long follicular phases with a stronger immune response; see figure 5 for more details. In each phase, the time average $\bar{m}$ of m(t) is used to obtain $\bar{A}(t)$ (red) numerically and (blue) using the approximation in equation (2.11) and the parameter values in ([11], table 3). Note the logarithmic scale for x(t) in (a).

Figure 4a shows an example of cancer development x(t) in the complete model (2.10) for a single realization of random mutations m(t) and periodically varying $s_{m}(t)$ due to the menstrual cycle. Figure 4b shows the moment when the cluster of mutated cells crosses the moving extinction threshold $\bar{A}(t)$ around the age of 51.25 and progresses into cancer. Noteworthy is the rescue event approximately 5 months earlier, around the age of 50.85; see also ([11], figure 2) for more examples of rescue events in the complete model.

**Figure 5.**
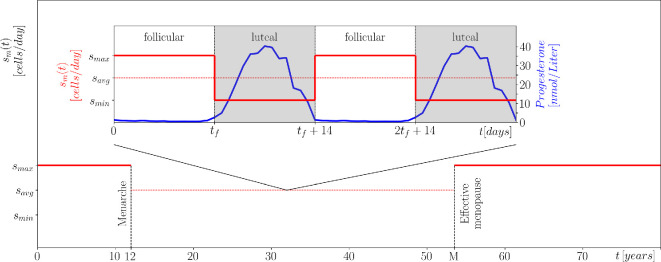
The immune response s(t) in equation (3.1) plotted over the lifetime of a woman. The inset shows (red) the assumed changes in immune response along with (blue) the changes in progesterone levels during the menstrual cycle [79] that occur between the age of 12 (the average age of menarche) and the age of effective menopause M. $s_{m}(t)$ is reduced during the (grey) luteal phase of the cycle due to the higher levels of progesterone [64,65,78]. M is chosen by the combination of natural menopause and delayed menopause due to HRT; see the three cases in §3.2.2.

## 3. Breast cancer case study

In this section, we use our complete cancer development model to reproduce real cancer data and provide new insights into breast cancer development in women. This discussion should be seen as a proof of concept of how simple dynamic models with an extinction threshold can be used to provide a qualitative description of cancer development and identify the underlying mechanisms and critical points.

### 3.1. Breast cancer progression in mice: a comparison between models and data

As data on the progression of untreated breast cancer in women is extremely limited, we examine the ability of both extended models with an extinction threshold, ERM1 and ERM2, to reproduce two different datasets on the progression of untreated breast cancer in mice. To do this, we combine a shooting method to solve each model and a curve fitting algorithm to estimate the model parameters ([11], section 2.1). We show that ERM2 can closely reproduce both datasets and give realistic estimates of the carrying capacity, which must be higher than any of the data points, in both datasets.^7^ In contrast, ERM1 fails to reproduce dataset 1.

**Figure 6.**
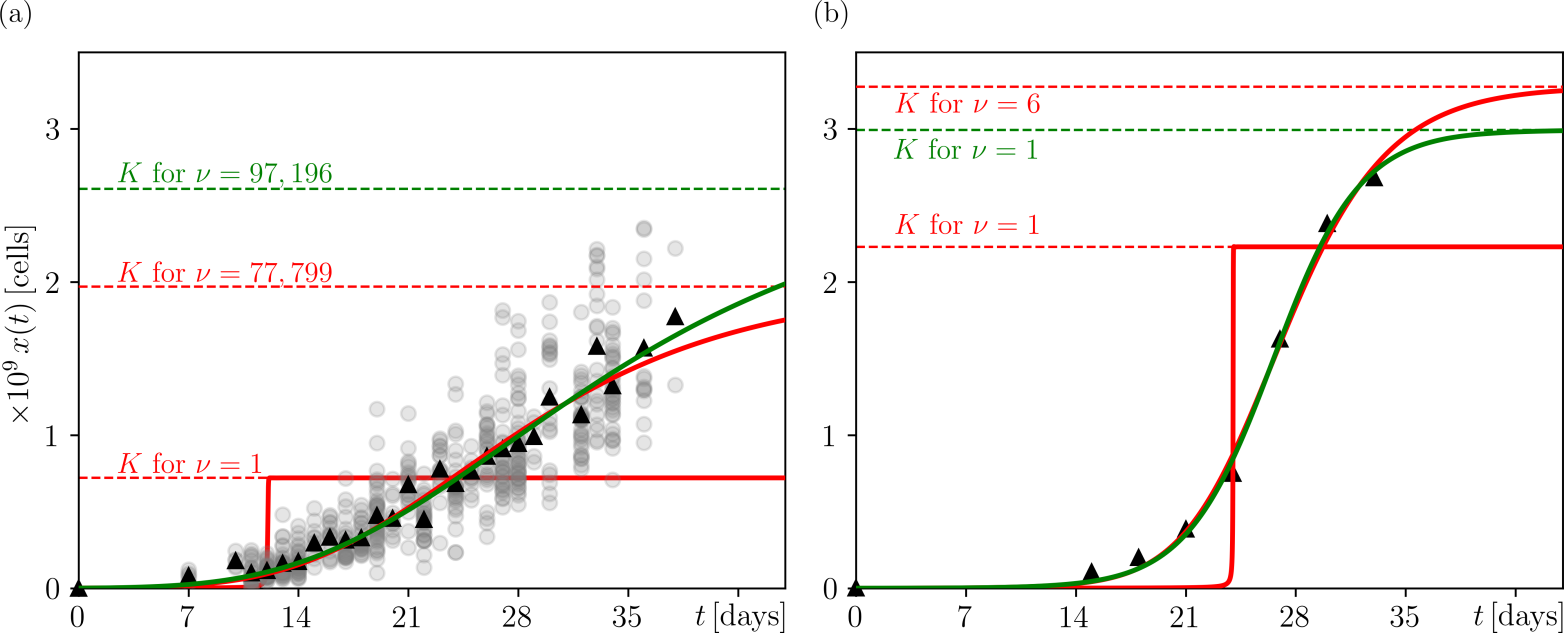
A comparison between real data on breast cancer progression in mice and dynamic models of cancer progression. (a) Dataset 1 from [44] shown as (circles) individual measurements of tumour size and (triangles) mean tumour size for each day, together with the best theoretical fits using (red curves) ERM1 and (green curve) ERM2. (b) Dataset 2 from [55] shown as (triangles) mean tumour size for each day, together with the best theoretical fits using (red curves) ERM1 and (green curve) ERM2. The model solutions start from the first data point at time t=0, the parameter values are listed in ([11], table 1), and the resulting carrying capacities are shown as dashed horizontal lines.

Dataset 1 from Vaghi *et al.* [44] consists of 583 data points from 65 severe combined immunodeficient (SCID) mice, collected over 38 days (grey dots in figure 6a). SCID mice exhibit severe immunodeficiency because of a lack of functional T and B lymphocytes [56]. However, they retain natural killer cells and preserve some anti-tumour activity, albeit at a much lower level than normal [57]. Each data point represents the tumour volume in one mouse at a point in time. Just for comparison with dataset 2, we also plot the average tumour volume at a point in time (black triangles in figure 6a). Dataset 2 from Cabeza *et al.* [55] consists of eight data points from 10 immunocompetent C57BL/6 mice, collected over 33 days (black triangles in figure 6a). Each data point represents the average tumour volume at a point in time.

Both datasets were obtained by injecting $x_{0}$ breast cancer cells into mice and monitoring the subsequent tumour volume progression without treatment until the mice died. To fit the models to the data, we convert the tumour volume to the number of cancer cells, assuming a cancer cell density of $10^{9}$ cells cm^−^${,}^{3}$ [58]. The model parameters that give the best fit are listed in ([11], table 1).

We begin with ERM1 in its factorized form (2.3). First, we set ν=1 to get the commonly used Volterra model and let the curve fitting algorithm estimate r,A and K. The best fit gives (red) step-like progression curves in figure 6a,b, which do not reproduce any of the properties of datasets 1 and 2, respectively. Second, we release ν and let the curve fitting algorithm estimate ν along with r,A and K. For dataset 1, the best fit gives ν≈77800 and a smoother (red) progression curve in figure 6a that only fails to reproduce the later stage of cancer progression. In particular, ERM1 underestimates the carrying capacity and returns a value of $K\approx 1.967\times 10^{9}$ cancer cells that is below some data points. This is inconsistent with the fact that a patient dies some time before the tumour reaches its carrying capacity [59]. For dataset 2, the best fit gives ν=6 and a smoother (red) progression curve in figure 6b that closely reproduces the cancer progression.

Next, we use ERM2 in (2.5) and let the curve fitting algorithm estimate its four parameters ν,r,μ and s. The best fit gives (green) progression curves in figure 6a,b, which are in very good agreement with datasets 1 and 2, respectively. In particular, both curves give realistic carrying capacities K.^8^ In figure 6a for dataset 1, the large value of ν=97196 indicates an asymmetric (practically Gompertzian) progression, which explains why dataset 1 cannot be fitted by ERM1. In figure 6b for dataset 2, the value of ν=1 indicates symmetric (logistic) progression, which explains why this dataset can also be fitted by ERM1 with larger ν.

Since the two groups of mice have significantly different immune responses, it is natural to ask whether this is captured by ERM2 as different extinction thresholds A. Comparing the results of the model for the two groups of mice, it can be seen that the predicted extinction threshold $A\approx 2\times 10^{2}$ cells for the immunodeficient SCID mice (dataset 1) is indeed significantly lower compared with $A\approx 3\times 10^{5}$ cells for the immunocompetent C57BL/6 mice (dataset 2); see ([11], table 1).

### 3.2. Breast carcinogenesis in women: reproducing unusual cumulative risk

The *cumulative risk of a particular cancer at age* t is the probability, expressed as a percentage, that a person will develop that cancer by age t [60]. The age-specific cumulative risk is the cumulative risk as a function of age t. The ability of a mathematical model to reproduce the cumulative risk of cancer depends crucially on its ability to capture the key mechanism(s) of carcinogenesis. Here, we use our complete cancer development model (2.10) to reproduce qualitatively different age-specific cumulative risks of different cancers.

**Figure 7.**
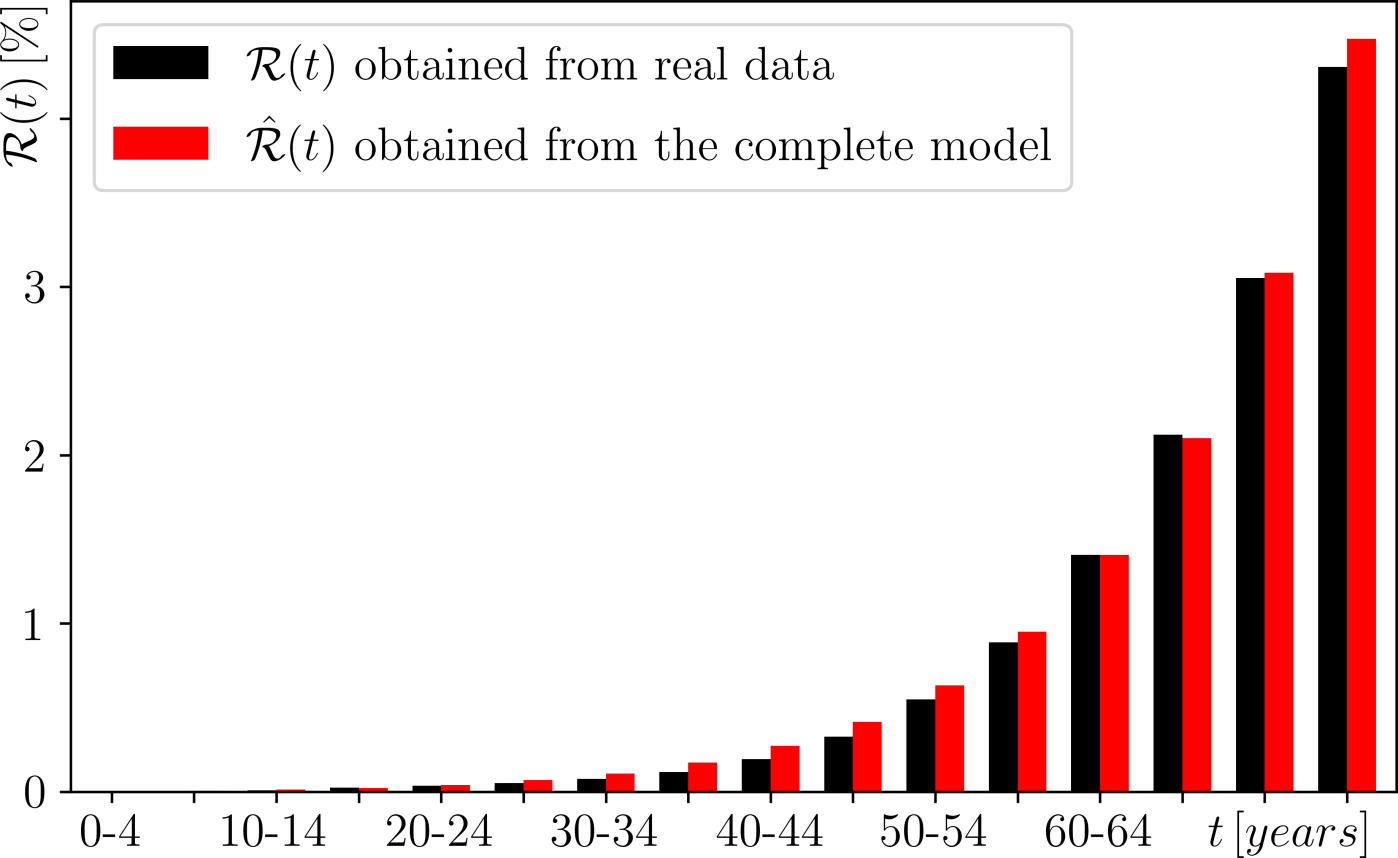
The age-specific cumulative risk of a woman developing colorectal cancer by a given age t. A comparison between the (black) actual risk R(t) obtained from real data in [61] and the (red) risk $\hat{R}(t)$ obtained from the complete model (2.10) with constant immune response $s(t)=s_{\text{max}}=const.$ We used the parameter values given in ([11], tables 2 and 3); refer to ([11], section 2.2.4) for more details.

**Figure 8.**
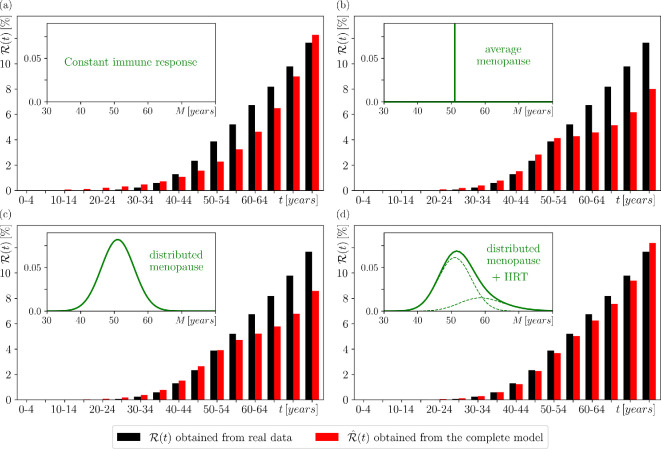
The age-specific cumulative risk of a woman developing breast cancer by a given age t. A comparison between the (black) actual risk R(t) obtained from real data in [61] and the (red) risk $\hat{R}(t)$ obtained from the complete model (2.10) with four different forms of immune response s(t). (a) A constant immune response with $s(t)=s_{\text{max}}=const.$ gives the (red) risk $\hat{R}(t)$, which does not match the (black) actual risk R(t). (b) A periodically varying immune response (3.1) between different levels during different phases of the menstrual cycle, with a fixed age of menopause M=51 years, gives good agreement with the (black) actual risk R(t) only up to the age band 50−54. (c) A periodically varying immune response (3.1) with normally distributed M gives a better agreement with the (black) actual risk R(t) but still underpredicts R(t) within the age bands 50−75. (d) A periodically varying immune response (3.1) with M that is normally distributed and then delayed for approx. 26% of women due to HRT closely reproduces the (black) actual risk R(t). We used the parameter values in ([11], tables 2 and 3).

The actual age-specific cumulative risk, which we denote by R(t), can be estimated from *age-specific incidence rate data*: the ratio of people diagnosed with a particular cancer to the number of all people in different age groups. To highlight different mechanisms of cancer development, we use the age-specific incidence rates of colorectal cancer and breast cancer in women in Ireland, obtained from the National Cancer Registry Ireland [61]. We then use these incident rates to calculate the R(t), shown in black for colorectal cancer in figure 7 and breast cancer in women in figure 8; see [62] and ([11], section 2.2) for more details.

The actual age-specific cumulative risk of colorectal cancer in women, (black) R(t) in figure 7, appears to increase exponentially with age ([11], figure 4). However, this is not the case for breast cancer in women (black) R(t) in figure 8. The striking feature of the actual age-specific cumulative risk of breast cancer in women is a (seventh-order) polynomial increase up to the age of 44−50, after which it changes and becomes linear ([11], figure 5). This is in contrast to not only colorectal cancer, but also the vast majority of cancers, which appear to show exponential-like increase in age-specific cumulative risk across all age groups; for example, see ([11], figure 6]) for blood cancer and brain cancer in women.

We denote the *theoretical age-specific cumulative risk* by $\hat{R}(t)$. $\hat{R}(t)$ is obtained by a combination of Monte Carlo simulations of our complete model (2.10) and a fitting algorithm, both of which are described in ([11], sections 2 and 3). To gain new insights into the different mechanisms that underlie the development of different types of cancer, we consider two cases: (i) a fixed immune response, and (ii) a time-varying immune response. The model parameters that give the best fits $\hat{R}(t)$ to the actual age-specific cumulative risks R(t) are listed in ([11], table 2). The results are robust in the sense that they persist when the model parameters, such as the immune response strength s and mutation probability $p_{0}$ are changed; see ([11], section 2.2.4) for a basic parameter sensitivity analysis.

#### 3.2.1. Typical age-specific cumulative risk of most cancers: fixed immune response

We start with a constant immune response,


$$s_{m}(t)=s_{\text{max}}=const.,$$

which gives rise to a fluctuating extinction threshold (2.11) due to mutations m(t) alone.

The fixed immune response model predicts an age-specific cumulative risk $\hat{R}(t)$ that increases exponentially with age. This exponential increase is in excellent agreement with the (black) actual risk R(t) for colorectal cancer in women in figure 7, and appears to be consistent with the age-specific cumulative risk for many other cancers ([11], figure 6). However, this $\hat{R}(t)$ does not capture the unusual age-specific cumulative risk of breast cancer in women. In figure 8a, the (red) $\hat{R}(t)$ overestimates the (black) R(t) below the age of 35 and underestimates it above the age of 35. Alternatively, we can get very good agreement between $\hat{R}(t)$ and R(t) below the age of 50−54, where we observe a polynomial increase, at the expense of strong disagreement above the age of 50−54, where there is a change and the increase becomes linear ([11], figure 4). This raises the question: what important process(es) related to breast cancer in women are missing from the model? We address this question in the next section.

#### 3.2.2. Unusual age-specific cumulative risk of breast cancer in women: time-varying immune response

We now allow the immune response to vary over time to explain the unusual age-specific cumulative risk of breast cancer in women. This results in a moving extinction threshold due to a combination of a time-varying immune response, $s_{m}(t)$ and random mutations, m(t).

The change in the increase of R(t) from polynomial to linear occurs around the age of 44−50 ([11], figure 4). It coincides with the menopause, which begins between the ages of 45 and 55 [63], when the natural menstrual cycle stops. This coincidence is the first indication that the menstrual cycle plays an important role in the development of breast cancer in women. Second, there is evidence that higher levels of *progesterone* during the luteal phase of the menstrual cycle weaken the immune system [64–66] and stimulate the growth of cancer cells [67]. While immunosuppression may be general [68] or specific to breast cancer [69], biological studies suggest that higher levels of progesterone during the luteal phase have a particularly strong effect on the mammary gland and therefore breast cancer, but not necessarily other cancers in women [70]. It has even been suggested that progesterone is the main driver of breast cancer risk during the menstrual cycle [71]. Third, there is evidence that a shorter menstrual cycle increases the risk of breast cancer [72–74]. These indications lead us to include in the model the effects of varying progesterone levels during the menstrual cycle and, for some women, during menopausal treatment.

The menstrual cycle is made up of different phases: the *luteal phase* lasts on average 14 days with little variation [75], *menstruation* lasts on average 5 days, the *follicular phase* also lasts on average 14 days but is much more variable [76] and *ovulation* lasts less than 24 hours. The total cycle length is reported to be between 19 and 44 days [77].

For the model, we assume that menstruation begins at the age of 12, and simplify the menstrual cycle into two phases: a fixed luteal phase of 14 days [75], and a variable follicular phase whose length $t_{f}$ is normally distributed with a mean $\mu_{f}=14$ days and a standard deviation $\sigma_{f}=2.4$ days [76]. We then use the fact that there is a noticeable increase in progesterone levels during the luteal phase [78], illustrated by the blue curve in the inset of figure 5. There is evidence that this increase weakens the immune response, $s_{m}$, and may increase the per capita growth rate of cancer cells, νr. We propose that the dominant effect is a weaker immune response $s_{m}$ during the luteal phase.^9^ Specifically, we assume that the immune response in the complete model (2.10) changes as follows during a woman’s lifetime:


(3.1) $$\begin{array}{r} s_{m}(t)=\left\{ \begin{array}{ll} s_{\text{max}} & \text{for}\;\;0<t<12,\\ s_{\text{min}} & \text{in luteal phases while}\;\;12\leq t<M,\\ s_{\text{max}} & \text{in follicular phases while}\;\;12\leq t<M,\\ s_{\text{max}} & \text{for}\;\;t>M, \end{array}\right. \end{array}$$

where M denotes the age of ‘effective’ menopause. This $s_{m}(t)$ is shown in red in the inset of figure 5. Next, we compare the actual age-specific cumulative risk of breast cancer in women obtained from real data, R(t), with $\hat{R}(t)$ obtained from the complete model (2.10) for three different cases of time-varying immune response (3.1). The model parameters are listed in ([11], table 3), where some of them are chosen using sparse data on the progression of untreated breast cancer in women ([11], section 3).

*Case 1: Average menopause*. In figure 8b, we use the average age at menopause and set M=51 years for all women; see the inset. The (red) $\hat{R}(t)$ closely reproduces the polynomial increase of the (black) R(t) below the age of 50−54, and shows a distinct change around 50−54 due to menopause. However, despite an improvement over the fixed immune response model, the (red) $\hat{R}(t)$ underestimates the (black) R(t) above the age of 50−54.

*Case 2: Distributed menopause*. In figure 8c, we use the age distribution at the onset of menopause, which is M is normally distributed with a mean $\mu_{M}=51$ years and a standard deviation $\sigma_{M}=4.86$ years [63,80]; see the inset. The (red) $\hat{R}(t)$ closely reproduces the polynomial increase of the (black) R(t) below the age of 50−54, shows a less distinct change around 50−54 due to menopause, but still underestimates the (black) R(t) above the age of 50−54. Although the model is getting closer to the actual data, it is still missing some of the necessary ingredients to achieve a very good agreement.

*Case 3: Distributed menopause with HRT*. It turns out that the missing ingredient is HRT. After the menopause, the body naturally stops producing hormones, including progesterone and oestrogen. HRT reduces menopausal symptoms by replacing the natural hormones with synthetic ones so the progesterone changes continue for the duration of HRT. Based on the data in [81,82], our model assumes that 26% of women use HRT, and that their progesterone changes continue past natural menopause for a period of time that has a gamma distribution with a mode of 6 years and a standard deviation of 4.8 years ([11], section 2.2.5). In figure 8d, we take HRT into account. Specifically, M is first drawn from the normal distribution of the onset of menopause and then, in 26% of cases, extended by the duration of HRT, which is drawn from the gamma distribution of HRT; see the inset. The results of the simulations show that the (red) $\hat{R}(t)$ now closely reproduces the (black) R(t) at all ages.

#### 3.2.3. Effects of menstrual cycle length on breast cancer risk: time-varying immune response

In addition to being able to reproduce the actual age-specific cumulative risk of different cancers, our complete cancer development model (2.10) with time-varying immune response (3.1) naturally accounts for the observation in [83] that the cumulative risk of breast cancer for women with cycles shorter than 25 days is 1.86 times higher than for women with cycles between 25 and 31 days.

**Figure 9.**
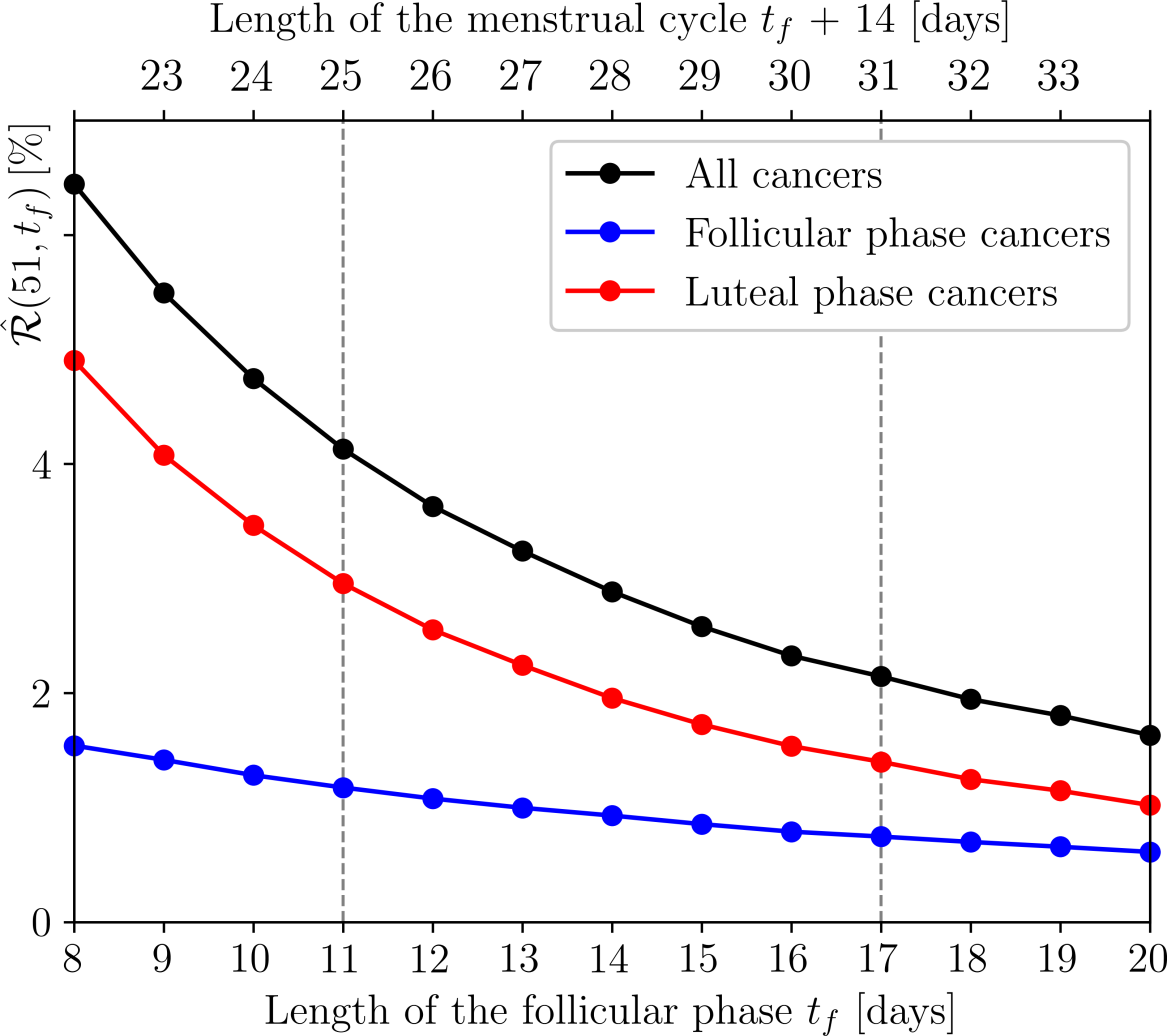
(Black) The model's cumulative risk of a woman developing breast cancer by the age of 51, for *Case 3* of §3.2.2, depending on follicular phase length $t_{f}$ and menstrual cycle length. The length of luteal phase length is fixed at 14 days. (Red and blue) The different risks of developing breast cancer during the luteal and follicular phases of the cycle, respectively. We use the parameter values in ([11], tables 2 and 3).

To show this, we consider *Case 3* of §3.2.2, which gives very good agreement with the real data. We then examine in figure 9 how the model’s simulated risk of developing breast cancer by the age of 51, that is $\hat{R}(51,t_{f})$, changes with the length $t_{f}$ of the much more variable follicular phase, while keeping the luteal phase at 14 days. Not only does the simulated risk $\hat{R}(51,t_{f})$ decrease significantly with increasing follicular phase length and thus menstrual cycle length, but it also predicts a 1.75-fold increase in risk for women with cycles between 22 and 25 days compared with women with cycles between 25 and 31 days. This prediction is in very good agreement with the observation in [83]. The model also shows that the greater proportion of transitions to cancer occur during the luteal phase when the progesterone levels are higher and the immune response is weaker. This proportion ranges from 76% for an 8 day follicular phase to 63% for a 20 day follicular phase, as shown by the red and blue curves, respectively, in figure 9. Thus, it may very well be that progesterone is the main driver of breast cancer risk during the menstrual cycle, as suggested in [71]. We refer to ([11], section 2.2.6) for more details.

## 4. Conclusions and outlook

In the context of cancer development, we define an extinction threshold as a critical size of a cluster of mutated cells below which the cluster can be eliminated by the immune system alone, and above which the cluster develops into cancer. We then construct a simple dynamic model to demonstrate that an extinction threshold is an essential component of cancer development, as evidenced by very good agreement with a wide range of real cancer data. The model incorporates random mutations, deterministic growth of mutated cells and an immune response that eliminates mutated cells. An important feature of the model is that the extinction threshold can move over time to reflect changes in the immune response, or other cancer-related processes, due to seasonal variations, viral infections, the menstrual cycle, immune deficiencies, immunotherapy, ageing, etc.

We began by introducing the defining feature of cancer progression: an asymmetry in which small cancers grow at the same rate or faster than large cancers saturate as they approach their carrying capacity. This allowed us to identify the limitations of classical extinction threshold models from population biology in reproducing cancer progression, and to derive a new model that overcomes these limitations. We then showed that the new model accurately reproduces a wide range of real-world cancer data, from the progression of breast cancer in mice to the population-level age-specific cumulative risk of different cancers in humans.

While we believe that this model can be applicable to a wide variety of cancers, we have chosen to focus on breast cancer for a number of reasons. First, breast cancer is one of the most common cancers in the world. Second, breast cancer in women has an unusual age-specific cumulative risk that appears to increase polynomially up to the age of menopause and linearly thereafter, which differs from exponential-like increase across all ages for many other cancers. Third, this difference has not been fully understood in terms of the underlying dynamics of cancer development. To the best of our knowledge, this is the first report on the prediction of actual cancer development and age-specific cumulative risk using dynamic models with an extinction threshold. We have shown that the concept of a moving extinction threshold, which reflects the different immune response at different phases of the menstrual cycle and HRT, can explain the unusual age-specific cumulative risk of breast cancer in women. In addition, our results are consistent with the observations that the risk of breast cancer is higher in women with shorter menstrual cycles and that combined HRT with oestrogen and progesterone increases the cumulative risk of breast cancer in women [84–86]. The latter observation led to a debate about the benefits of combined HRT versus the increased risk of cancer. Our model could make a valuable contribution to this debate by providing new insights into the effects of combined HRT; note the difference between (red) $\hat{R}(t)$ in figure 8c,d. In particular, it provides a basis for development of more detailed models to better quantify the effects of HRT on breast cancer risk in women. More generally, our approach can be applied to other cancer scenarios where certain threshold parameters change over time, leading to atypical cumulative risks. These changes can occur for biological reasons and in temporal patterns that are similar to or different from breast cancer in women.

From the point of view of mathematical modelling, recently there has been much interest in the theory of tipping points (critical transitions) [87] and evolutionary games for adaptive therapy of cancer [88]. It turns out that carcinogenesis in our model is an example of a tipping point induced by bounded noise: a point in time when random mutations cause a critical transition across the extinction threshold from a cancer-free state to the first stage of cancer. At the same time, our model of cancer development is simple enough for the techniques of evolutionary games and optimal adaptive therapy [16,89]. These features make our model a natural candidate for combined analysis using the techniques of tipping point theory and evolutionary games to explore new optimal cancer treatment strategies. Furthermore, the model can be easily extended to capture more processes within the tumour microenvironment by adding separate equations governing the dynamics of the immune cells [90] and the tumour stroma [43]. Other worthwhile extensions include treatment strategies that exploit the possibility of rescue events, and the combination of our dynamic mutation model with a Markov chain approach to mutation accumulation [18].

## Data Availability

Code is available in Zenodo [91]. Supplementary material is available online [11].

## Footnotes

^1^
Linear increase does not accelerate, polynomial accelerates and exponential accelerates faster than polynomial.

^2^
The number of cancer cells is a natural number. However, for large numbers of cells, it is convenient to model them using real numbers.

^3^
A per capita rate of change is the rate of change for each cell, or per unit of population and is given by (dx/dt)/x.

^4^
This process is similar to harvesting in population biology.

^5^
Since random mutations have a positive expected value, $s_{m}(t)=s(t)+\langle \xi (t+\Delta t)-\xi (t)\rangle /\Delta t$, where s(t) is the immune response in the absence of mutations and ⟨ξ(t+Δt)−ξ(t)⟩ is the slowly - varying expected value of new mutations during the time interval Δt.

^6^
Note that the term in square brackets is multiplied by a factor of 1 day, which is not shown.

^7^
In this section, in ERM2, we use a constant immune response s(t)=s=const. to analyse progression above the threshold.

^8^
Unlike ERM1, ERM2 has no prescribed A and K, and their values must be obtained as equilibrium points for ERM2.

^9^
The approximation (2.11) shows that an increased per capita growth rate νr, due to higher progesterone levels during the luteal phase, has a similar effect to a weaker immune response $s_{m}(t)$—both lower the extinction threshold during the luteal phase.
